# Beyond the Needle: Innovative Microneedle-Based Transdermal Vaccination

**DOI:** 10.3390/medicines12010004

**Published:** 2025-02-07

**Authors:** Hiep X. Nguyen

**Affiliations:** Faculty of Pharmacy, Phenikaa University, Yen Nghia, Ha Dong, Hanoi 12116, Vietnam; hiep.nguyenxuan@phenikaa-uni.edu.vn; Tel.: +1-404-820-4015

**Keywords:** microneedles, vaccination, transcutaneous immunization, classifications, challenges

## Abstract

Vaccination represents a critical preventive strategy in the current global healthcare system, serving as an indispensable intervention against diverse pathogenic threats. Although conventional immunization relies predominantly on hypodermic needle-based administration, this method carries substantial limitations, including needle-associated fear, bloodborne pathogen transmission risks, occupational injuries among healthcare workers, waste management issues, and dependence on trained medical personnel. Microneedle technology has emerged as an innovative vaccine delivery system, offering convenient, effective, and minimally invasive administration. These microscale needle devices facilitate targeted antigen delivery to epidermal and dermal tissues, where abundant populations of antigen-presenting cells, specifically Langerhans and dermal dendritic cells, provide robust immunological responses. Multiple research groups have extensively investigated microneedle-based vaccination strategies. This transdermal delivery technique offers several advantages, notably circumventing cold-chain requirements and enabling self-administration. Numerous preclinical investigations and clinical trials have demonstrated the safety profile, immunogenicity, and patient acceptance of microneedle-mediated vaccine delivery across diverse immunization applications. This comprehensive review examines the fundamental aspects of microneedle-based immunization, including vaccination principles, transcutaneous immunization strategies, and microneedle-based transdermal delivery—including classifications, advantages, and barriers. Furthermore, this review addresses critical technical considerations, such as treatment efficacy, application methodologies, wear duration, dimensional optimization, manufacturing processes, regulatory frameworks, and sustainability considerations, followed by an analysis of the future perspective of this technology.

## 1. Introduction

### 1.1. Introduction of Vaccination

Immunization represents a critical therapeutic intervention and revolutionary advancement in modern preventive medicine for combating infectious diseases. Vaccine development stands as an unprecedented milestone in public health achievements [1,2]. Statistical analysis from the World Health Organization demonstrates that immunization protocols prevent 2–3 million fatalities annually, with the potential to avert 3.5–5 million deaths per year [3]. Nevertheless, infectious diseases continue to claim approximately 15 million lives globally per annum [4]. Current vaccination strategies provide protection against more than 25 potentially lethal pathogens while facilitating population-level immunity, which necessitates the immunization coverage of approximately 70% of the demographic groups [5]. This extensive deployment has led to the elimination, containment, or diminished significance of numerous infectious diseases, contributing to enhanced human longevity. Epidemiological analyses spanning 1994–2013 reveal that immunization protocols averted roughly 732,000 premature mortalities and 320 million disease symptoms, yielding economic benefits exceeding USD 295 billion in healthcare expenditure reduction [6].

Vaccines comprise biological preparations incorporating viable or attenuated antigenic components that elicit immunological responses. These formulations contain modified microorganisms in weakened or inactivated forms, associated toxoids, or surface antigens [7]. Such compositions trigger immunological cascades, promoting antibody generation against specific pathogens while averting disease symptoms [8,9]. Current vaccine classifications include whole-pathogen formulations, subunit preparations, genetic constructs, and viral vectors, with emerging types, including nucleic acid-based approaches (DNA/RNA vaccines) under development [10]. Immunological responses to vaccine administration are demonstrated through dual mechanisms. The primary phase involves innate immunity, delivering non-specific defense through specialized cellular components, including natural killer cells, macrophages, and dendritic cells. Subsequently, adaptive immunity mobilizes T and B lymphocytes for antibody production, culminating in memory cell formation that confers sustained immunological protection, as exemplified in smallpox eradication [11].

Economic analyses from 2011 to 2020 indicate that immunization campaigns across 94 low and middle-income nations required USD 62 billion, wherein distribution networks and supply chain management consumed 54% and 6% of expenditures, respectively [4]. A significant operational challenge involves maintaining stringent temperature-controlled environments, with cold-chain infrastructure demanding annual global expenditures of USD 200–300 million. Among the five billion vaccine doses administered annually worldwide, three billion utilize needle-based delivery systems [12]. Recent vaccination protocols face multiple challenges: suboptimal bioavailability via mucosal routes, occupational hazards for healthcare practitioners with needlestick injuries affecting 56.2% of personnel during their professional tenure [13], and public resistance to vaccination, partially attributed to needle phobia and safety concerns [14,15].

Optimal vaccine formulations should demonstrate safety profiles, therapeutic efficacy, thermal stability, and administration simplicity while generating sustained immunological responses. The World Health Organization prioritizes vaccine development and mass immunization programs targeting influenza, HIV/AIDS, tuberculosis, Hepatitis B, Ebola, and Zika viruses [16]. Several vaccine products have achieved commercial success, generating annual revenues exceeding one billion dollars [17].

### 1.2. Route of Vaccine Delivery

**Figure 1:**
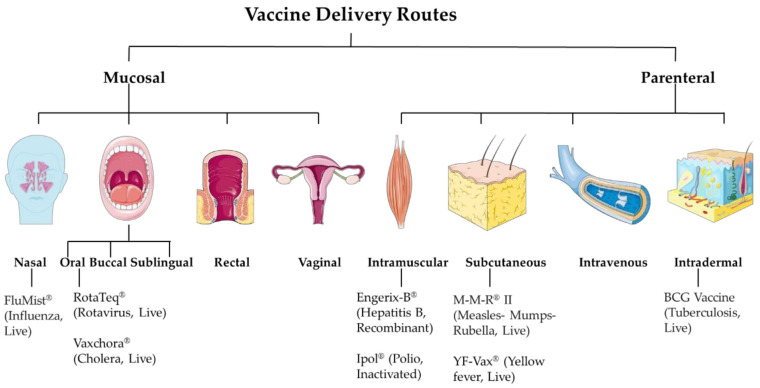
Route of vaccine delivery. Image reprinted with permission from [19], 2021, *Micromachines*.

Immunization strategies involve diverse administration routes, fundamentally affecting vaccine efficacy and immunological outcomes [18]. The strategic selection of delivery pathways remains contingent upon vaccine composition, spanning live attenuated formulations to advanced mRNA vaccines while considering the requisite balance between mucosal and systemic immunological responses [19]. Present vaccination methodologies are divided into two principal categories: parenteral and mucosal delivery systems (Figure 1) [12,19,20]. While conventional parenteral administration predominantly employs intramuscular or subcutaneous routes utilizing standard needle-based delivery systems, select immunizations, notably rotavirus, adenovirus, and typhoid formulations, necessitate oral administration [21].

Mucosal vaccination strategies specifically target mucosa-associated lymphoid tissue (MALT), incorporating innate and adaptive immune components [22]. These approaches present numerous advantages: enhanced patient acceptability, reduced dependence on healthcare practitioners, the minimization of sharps-related hazards, and the concurrent stimulation of mucosal and systemic immunity [23]. Intranasal delivery, exemplified by FluMist Quadrivalent^®^ (MedImmune, LLC, Gaithersburg, MD, USA), specifically engages nasal-associated lymphoid tissues, facilitating robust innate and humoral immune responses [24]. Alternative mucosal routes include oral, buccal/sublingual, rectal, and vaginal delivery systems. Oral administration induces comprehensive immune responses through Peyer’s patch activation and intestinal MALT stimulation [25]. Despite favorable safety profiles and administration simplicity, these approaches face significant challenges, including gastrointestinal degradation and required high antigen doses. Moreover, live attenuated oral vaccines present potential virulence reversion risks, particularly concerning immunocompromised populations [19]. The buccal and sublingual regions contain specialized antigen-presenting cells capable of triggering adaptive immune responses with reduced antigen requirements compared to oral delivery [26]. While rectal and vaginal routes present viable alternatives for specific pathogens, patient acceptance remains a significant consideration [19].

Parenteral administration routes maintain predominance in vaccination protocols, offering superior bioavailability through gastrointestinal bypass. Primary methodologies include intramuscular, subcutaneous, and intradermal injections. The integumentary system functions simultaneously as a protective barrier and immunological organ, providing substantial immune response capabilities [27].

Intramuscular vaccination, particularly relevant for inactivated and mRNA-based formulations, generates robust systemic immunity despite lower concentrations of antigen-presenting cells compared to cutaneous tissue [9,19]. Intradermal delivery, characterized by abundant immune cell populations, demonstrates equivalent immunogenicity with substantially reduced dosing requirements, achieving comparable responses with 20% of standard doses [28]. Subcutaneous administration remains primarily indicated for live-attenuated vaccines [19]. While intravenous delivery offers maximal bioavailability, conventional immunization protocols generally exclude this route due to rapid clearance kinetics and safety considerations.

Current vaccination challenges include multiple dimensions. Injectable formulations necessitate trained medical personnel and generate biohazardous waste. World Health Organization data indicate that unsafe injection practices annually contribute to substantial HIV and Hepatitis transmission rates. Needle anxiety, affecting 20–50% of adolescents and 20–30% of young adults, significantly impacts vaccination compliance and may precipitate severe physiological responses [29]. Furthermore, stringent cold-chain requirements from manufacturing through administration incur substantial costs, approximately USD 200–300 million annually. WHO reports indicate that 50% of global vaccine wastage is predominantly attributable to cold-chain failures in developing regions [30]. Emerging technologies address these limitations through innovative delivery platforms, including needle-free injection systems, microneedle arrays enabling painless administration while circumventing cold-chain requirements [19], and transcutaneous immunization methodologies. Advanced delivery systems, including gene guns and electroporation devices, demonstrate particular efficacy in nucleic acid vaccine delivery [31], with devices such as PharmaJet Tropis achieving regulatory approval for COVID-19 DNA vaccine administration in India [32,33].

### 1.3. Classification of Vaccines

Present human vaccination strategies involve four principal categorical classifications: attenuated viable vaccines, inactivated pathogenic preparations, constituent-based immunological agents (i.e., nucleic acid-based formulations such as DNA and RNA components, protein subunits, and virus-like particles (VLPs)), and detoxified bacterial toxin vaccines. These immunoprophylactic approaches can be systematically dichotomized into whole-pathogen preparations (comprising both attenuated and inactivated formulations) and subunit-based vaccine technologies (derived from specific pathogenic components). Vaccine-preventable viral pathologies of significant public health concern include hepatotropic infections (Hepatitis A and B viruses), acute respiratory diseases predominantly affecting pediatric populations (measles virus), and systemic infections characterized by distinctive exanthematous presentations (rubella virus) [11]. The therapeutic treatment targeting these and related infectious diseases demonstrates remarkable diversity in immunological approaches, as exemplified by the oral poliovirus vaccine (attenuated viable preparation), Hepatitis A and B prophylactic formulations (inactivated viral particles), SARS-CoV-2 immunization (mRNA technology), human papillomavirus prevention (VLP-based vaccine), and tetanus prophylaxis (detoxified bacterial exotoxin) [34,35].

## 2. Roles of Skin in Vaccination

**Figure 2:**
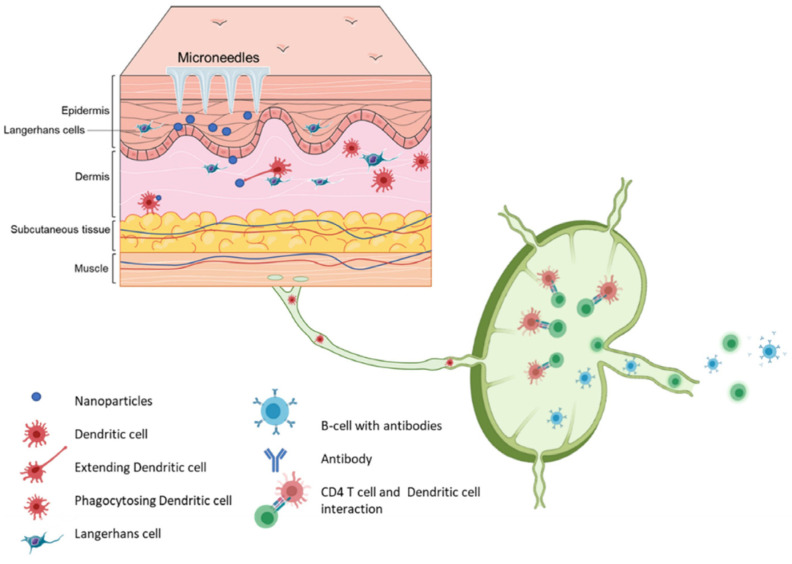
The structure and immunological network of cutaneous tissue. The structural arrangement progresses from the external stratum corneum through the epidermis to the dermis. The cutaneous immune surveillance system contains epidermal keratinocytes and Langerhans cells, dermal dendritic cells, fibroblasts, and mast cells within the dermis, complemented by T and B lymphocytes residing in subcutaneous lymphoid tissue. Langerhans cells and dermal dendritic cells serve as primary antigen-presenting cells, facilitating antigen capture and presentation to lymphocytes, thereby inducing robust humoral and cellular immune responses. Image reprinted with permission from [19], 2021, *Micromachines*.

The integumentary system functions as a sophisticated immunological barrier, providing both innate and adaptive immune responses [36]. The structure comprises distinct strata: the outermost stratum corneum, followed by the epidermis, dermis, and hypodermis, which collectively contain an array of immunocompetent cells, including keratinocytes, Langerhans cells (LCs), dendritic cells (DCs), T-lymphocytes, and macrophages (Figure 2) [37]. The cutaneous immune system operates through multiple sophisticated mechanisms. Keratinocytes execute antigen recognition via pattern recognition receptors (PRRs), subsequently initiating the secretion of immunomodulatory molecules, including cytokines, chemokines, and antimicrobial peptides. Within this complex network, LCs and dermal DCs function as specialized antigen-presenting cells (APCs), facilitating antigen capture and subsequent presentation to T-lymphocytes [37]. Following antigenic stimulation, LCs undergo maturation, process antigenic material, and travel to skin-draining lymph nodes, where these cells activate both CD4^+^ T helper lymphocytes and CD8^+^ cytotoxic T lymphocytes [38]. This cascade culminates in B-lymphocyte activation, promoting antibody secretion and memory cell formation, thereby establishing robust humoral and cell-mediated immunity [39].

Despite the abundant presence of immunological cells rendering the skin an optimal vaccination site, the stratum corneum presents a barrier, permitting penetration exclusively to lipophilic and low-molecular-weight compounds [40]. Transdermal vaccination strategies demonstrate marked advantages over conventional intramuscular administration, potentially enabling dose reduction while simultaneously enhancing immunogenicity and reducing manufacturing costs. Nevertheless, traditional intradermal delivery methods present technical challenges and cause significant discomfort, resulting in limited clinical implementation [41]. Addressing these limitations has driven the development of innovative delivery methods, notably including microneedle technology.

## 3. Skin Vaccination Strategies

Vaccine administration via the intradermal route, traditionally accomplished through hypodermic needle injection, remains the gold standard delivery methodology [42]. This approach demonstrates superior immunogenic responses compared to intramuscular or subcutaneous administration, attributed to the enhanced concentration of immunological components within skin tissue [43,44]. Notably, comparable immunological outcomes can be achieved with reduced antigen doses through intradermal delivery [43,44,45]. Nevertheless, conventional hypodermic needle-based administration presents numerous clinical challenges: needle phobia, pathogen transmission through needle reuse, variable delivery precision, occupational needle-stick exposure, biomedical waste management concerns, and adverse reactions [20,46,47]. These limitations are especially significant in pediatric populations that receive multiple immunizations [48].

Recent research has witnessed the emergence of alternative vaccination strategies, including both noninvasive and minimally invasive skin delivery techniques [49]. Noninvasive methodologies, including ballistic jet delivery, thermal ablation techniques, sonophoresis, and electropermeabilization, aim to circumvent needle-based delivery while enhancing transdermal permeation [50]. Minimally invasive approaches, exemplified by microneedle array systems such as BD Soluvia^TM^ and Onvax^TM^, facilitate reproducible and efficacious vaccine delivery while minimizing patient discomfort and reducing dependence on healthcare professionals [49]. In addition, investigations have explored DNA vaccine administration through intradermal tattooing techniques [51]. Despite these innovations, alternative delivery methods frequently necessitate sophisticated instrumentation, may induce patient incompliance, and demonstrate variable delivery consistency or suboptimal vaccine penetration [52,53].

## 4. Microneedle-Mediated Vaccination Strategies

**Figure 3:**
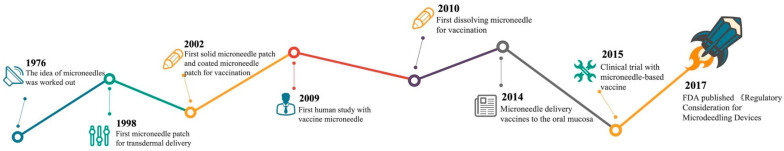
History of microneedle development. Image reprinted with permission [57], 2023, *Pharmaceutics*.

The pioneering concept of microneedles emerged several decades ago, with systematic scientific investigation commencing in the mid-1990s (Figure 3) [54]. These microscale transdermal devices, ranging from 50 to 2000 μm in height, enable the efficient penetration of the stratum corneum barrier for therapeutic agent administration [55]. The fundamental principle underlying microneedle technology draws inspiration from Edward Jenner’s revolutionary intradermal vaccination strategy against smallpox, strategically targeting the abundant population of antigen-presenting cells within the dermis [56].

**Figure 4:**
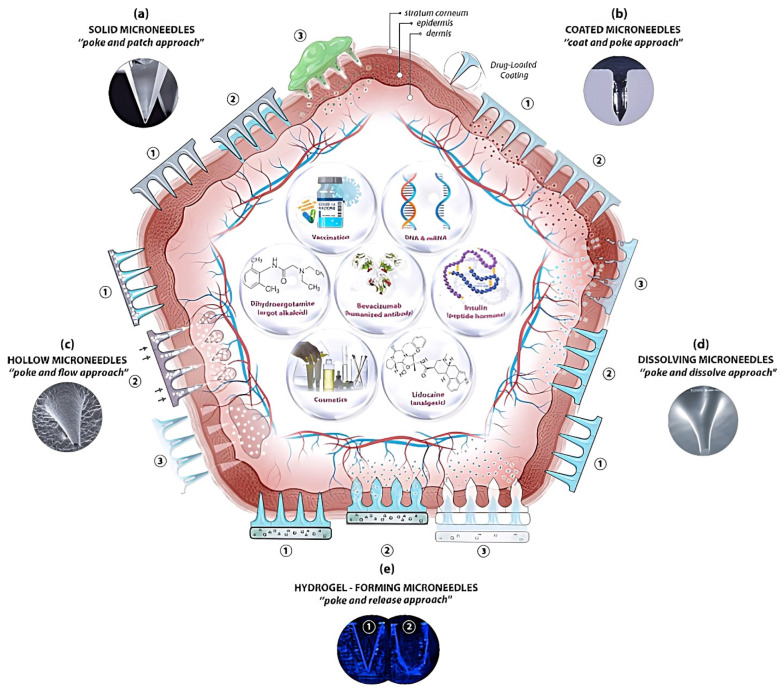
Various types of microneedles for transcutaneous immunization. Image reprinted with permission from [59], 2021, *Micromachines*.

Current microneedle designs include multiple structural configurations: solid matrices, hollow conduits, surface-modified variants, and bioresponsive dissolving systems (Figure 4). These distinct microneedle types offer differential advantages regarding manufacturing economics, scalability parameters, patient self-administration feasibility, and therapeutic release kinetics. The field has witnessed remarkable progression since the seminal investigations in 2002 [58], demonstrating successful clinical translation across diverse vaccine applications, such as influenza, rabies, human papillomavirus (HPV), and SARS-CoV-2 immunizations [9]. BD Soluvia™ (Becton Dickinson, NJ, USA) was the first FDA-approved microneedle system for vaccine delivery. The device incorporates a single hollow microinjection needle measuring 1.5 mm in length and is engineered to deliver the vaccine intradermally. The clinical trials demonstrated equivalent immunogenicity with reduced antigen dose compared to conventional IM injections. Significant regulatory milestones include the European Union’s authorization of Sanofi Pasteur’s Intanza^®^ in 2009 and the subsequent approval of Fluzone^®^ by United States regulators in 2011, culminating in the Food and Drug Administration’s publication of comprehensive regulatory guidelines in 2017 [57]. NanoPass Technologies’s MicronJet™ exemplifies an FDA-cleared single-use microneedle device featuring a triad of hollow microneedles with dimensions of 0.45 mm in length. Clinical investigations studied various vaccines, including influenza and polio antigens, and have demonstrated antigen dose reduction while maintaining therapeutic efficacy. The Hollow Microstructured Transdermal System (hMTS), developed by 3M, secured FDA clearance for transdermal drug delivery and incorporated an array of hollow microneedles. Nevertheless, regulatory approval for specific vaccine formulations utilizing this delivery system remains pending. Current development efforts targeting alternative microneedle types, specifically dissolving and coated microneedles, persist across various phases of clinical development. Despite promising immunological outcomes observed in human trials, these innovative devices await regulatory approval for vaccine delivery.

### 4.1. Solid Microneedles

Pioneering investigations established solid microneedles as the first generation of transcutaneous delivery systems, comprising metallic, silicon-based, and non-biodegradable polymeric microneedles [60]. These systems operate via a sequential “poke and patch” mechanism, whereby microscopic channels are initially created, followed by the topical administration of therapeutic or immunogenic formulations to the treated site [61,62]. The therapeutic agents subsequently permeate through these micropathways into the underlying dermal tissue [63,64]. Extensive preclinical investigations have demonstrated successful vaccine delivery utilizing solid microneedles across multiple immunological applications, such as diphtheria, influenza, Hepatitis B, malaria, and human papillomavirus (HPV). The microneedle-mediated vaccination exhibited superior immunogenicity compared to conventional parenteral administration routes. Microneedles deliver antigens directly to the epidermis and dermis layers, regions densely populated with dermal antigen-presenting cells, particularly Langerhans cells and dermal dendritic cells, resulting in more efficient antigen presentation and subsequent immune response activation. Furthermore, the microneedle-induced skin disruption creates localized microtrauma, enhancing the innate immune response and leading to more robust adaptive immunity development. Several studies have demonstrated that microneedle-based vaccination could achieve equivalent or superior immune responses using lower antigen doses compared to traditional injections. Notably, the Nanopatch^TM^ array system enhanced HPV vaccine immunogenicity, enabling adjuvant-independent administration [65].

The structural integrity of solid microneedles necessitates materials possessing substantial mechanical strength to facilitate stratum corneum penetration, primarily utilizing silicon, metallic compounds, and ceramic materials. Manufacturing methodologies include various microfabrication techniques, including dry and wet etching processes, laser ablation, and micro-molding approaches. Polymeric solid microneedles have been explored, albeit demonstrating inferior mechanical properties. The extensive investigation of solid microneedles as transcutaneous delivery platforms has yielded promising preclinical results for vaccine and therapeutic administration. Despite promising preclinical outcomes, solid microneedles present several technological limitations. These challenges include necessitating biphasic administration protocols [66], potential metallic residue deposition in cutaneous tissue [67], and suboptimal control over drug delivery kinetics [68]. These technological constraints have catalyzed the development of alternative microneedle designs, including coated and hollow microneedles.

### 4.2. Hollow Microneedles

Hollow microneedles represent miniaturized analogs of conventional hypodermic needles, functioning as microscale injection devices that facilitate therapeutic agent administration [54]. These devices incorporate internal channels or luminal spaces that enable the transport of liquid pharmaceutical formulations through the stratum corneum [69,70]. The hollow structure permits the administration of substantially greater volumes of active pharmaceutical ingredients with enhanced continuity compared to alternative microneedle configurations [71].

The fabrication of hollow microneedles predominantly employs materials, including metallic substrates, vitreous compounds, ceramic matrices, and silicon-based substances [69]. Manufacturing complexities arise from the inherent structural vulnerability of these microscale devices. Present fabrication methodologies leverage microelectromechanical system (MEMS) technologies, including laser-assisted micromachining, deep reactive ion etching processes, deep X-ray lithographic techniques, and wet chemical etching procedures for large-scale production [54]. Drug and vaccine administration via hollow microneedles proceeds through two primary mechanisms: pressure-driven fluid dynamics or passive molecular diffusion from integrated reservoirs [72]. Single-needle pressure-driven systems contrast with reservoir-based arrays, where the latter enables enhanced spatial coverage and superior drug delivery rates relative to conventional subcutaneous administration [73]. Uniform pressure distribution across the entire array remains crucial for preventing formulation leakage [74]. The typical applied pressure for vaccine delivery through hollow microneedles ranges from 5 to 20 psi, depending on the specific microneedle design and formulation viscosity [75]. This pressure range ensures effective delivery while maintaining patient comfort and tissue integrity. Regarding injection volumes, hollow microneedles typically accommodate the infusion of up to a few hundred microliters [54]. The infusion rates are normally quite low (50–300 nL/min) [76]. While hollow microneedles demonstrate broad applicability, their suitability varies based on several factors, including vaccine formulation viscosity, molecular size of the antigen, the required dose volume, and stability of the vaccine components.

Preclinical studies and human trials have demonstrated successful vaccine delivery using hollow microneedles across multiple therapeutic applications, including poliomyelitis, influenza, plague, and human papillomavirus immunizations [77,78]. Microneedle technology accommodates vaccine administration with or without immunological adjuvants, demonstrating the robust activation of both humoral and cellular immune responses, frequently at reduced dosages compared to traditional parenteral delivery [79,80]. Nevertheless, hollow microneedle systems present specific technical challenges, including lumen occlusion, formulation extravasation, and mechanical integrity concerns that may lead to needle fracture and associated safety implications [81]. The compact structure of dermal tissue can additionally influence drug release kinetics through microneedle channels [70]. This transdermal delivery platform presents a promising avenue for minimally invasive vaccine administration, particularly through targeted delivery to immunologically active dermal layers. Ongoing research endeavors remain essential to address existing technological limitations and optimize system performance.

### 4.3. Coated Microneedles

Coated microneedle technology involves solid microstructures functionalized with therapeutic agents through a solution or dispersion-based coating. The fabrication methodologies include various deposition techniques, including dip-coating processes, spray deposition, and precision ink-jet application [82,83]. Formulation optimization remains critical for diverse vaccine types to preserve antigenic efficacy and maintain stability during fabrication [84,85]. Standard formulation components incorporate viscosity-enhancing excipients, stabilizing agents, and immunological adjuvants [82,86,87]. The therapeutic application spectrum encompasses numerous vaccine candidates, including prophylactic formulations against influenza, human papillomavirus, chikungunya virus, West Nile virus, rotavirus, herpes simplex virus, Hepatitis C, bacillus Calmette-Guérin, Hepatitis B virus, measles, and poliomyelitis. Coated microneedles demonstrate notable advantages over conventional hypodermic delivery systems, offering rapid drug release, enhanced bioavailability, precise dosing capabilities, and versatility for both small and large-molecular-weight compounds [70].

Therapeutic loading capacity remains a fundamental limitation governed by coating dimensions and microneedle geometry [88]. Strategic approaches to overcome these limitations include array dimensional optimization, increased needle density, and the implementation of electrostatic layer-by-layer (LbL) coating methodologies [89,90]. These technological advances enhance vaccine delivery efficacy against viral pathogens and malignancies.

Critical challenges persisting in clinical translation include uneven drug distribution, limited loading capacity, and manufacturing scalability constraints. For instance, Kim et al. could coat up to 7–8 μg of inactivated influenza virus per five-microneedle array [91]. Advanced formulation development and innovative patch engineering remain imperative [70]. Clinical validation through Phase I trials conducted by Vaxxas Pty Ltd. has demonstrated promising outcomes for influenza vaccination [65,86]. The studies revealed excellent tolerability, safety, and enhanced immune responses with a six-fold dose reduction compared to conventional intramuscular administration. Additional benefits include dose-sparing potential and enhanced thermostability, eliminating cold-chain requirements. Specifically, thermal stability studies demonstrated the preservation of influenza vaccine coated on high-density microarray patches when stored at 40 °C [86].

### 4.4. Dissolving Microneedles

Water-soluble biocompatible and biodegradable polymers or saccharides serve as the fundamental constituents of dissolving microneedles. These transdermal delivery systems operate via a “poke and release” mechanism, whereby the microneedles undergo gradual dissolution within the cutaneous tissue following penetration, facilitating the sustained release and subsequent systemic absorption of incorporated therapeutic agents [92]. Dissolving microneedles demonstrate distinct advantages over their solid and hollow counterparts, notably through simplified fabrication processes and single-step patch application [11]. The manufacturing protocol employs micro-molding techniques, wherein the aqueous solutions of constituent materials undergo controlled solidification within the molds. The fabrication of dissolving microneedles employs diverse polymeric and saccharide-based materials, including polyvinylpyrrolidone, polyvinyl alcohol, hyaluronic acid, chitosan, polycaprolactone, carboxymethylcellulose, and poly(lactic-co-glycolic acid). Material selection necessitates the careful consideration of biocompatibility, aqueous solubility, and sufficient mechanical properties to achieve dermal penetration [70]. Furthermore, the encapsulation and solidification protocols for labile molecules, including proteins, peptides, and vaccines, demand moderate processing conditions to preserve biological activity [93].

Extensive research has revealed the effectiveness of dissolving microneedles in transdermal vaccine delivery against numerous infectious diseases and malignancies. Vaccine antigens can be incorporated either through direct encapsulation within the dissolving matrix or via the integration of antigen-loaded micro- and nanoparticulate systems [94]. These approaches provide enhanced vaccine stability and enable controlled antigen release kinetics [95]. The penetration efficiency and vaccine delivery capabilities of dissolving microneedles depend significantly on their geometric parameters, including needle morphology, length, tip diameter, base dimensions, and spatial distribution [96,97,98]. The optimization of these structural features enhances antigen-presenting cell activation and subsequent immunological responses.

Dissolving microneedles have demonstrated remarkable capabilities in maintaining vaccine antigen stability at ambient temperature conditions for extended durations, addressing cold-chain challenges for various antigens, including influenza, malaria, Hepatitis B, and COVID-19. In particular, storage stability assessments revealed the complete preservation of adenovirus vaccine immunopotency when formulated in dissolving microneedle arrays during exposure to accelerated conditions (40 °C, 75% relative humidity) for an 8–12 week duration. Moreover, vaccine viability remained uncompromised throughout a six-month evaluation period under intermediate storage conditions (25 °C, 60% relative humidity) [99]. Such stability enhancement stems from the incorporation of specialized excipients, including saccharides, amino acids, and polymeric stabilizers within the microneedle matrix [100]. Advanced dissolving microneedle designs have emerged to enhance delivery efficiency and user compliance, featuring innovations such as detachable needle tips that remain embedded post-insertion [101] alongside simplified applicator systems that eliminate requirements for specialized administration equipment [102]. The dissolving microneedles present compelling advantages for vaccine delivery, including enhanced patient acceptability, the elimination of biohazardous waste, and the potential development of thermally stable formulations that circumvent cold-chain requirements [41].

### 4.5. Swelling Microneedles

Hydrogel microneedles represent an innovative class of transdermal delivery systems comprising cross-linked hydrogels or super-swelling polymeric matrices [103]. These advanced delivery systems serve dual functions: facilitating drug administration through controlled swelling mechanisms and enabling diagnostic applications via the selective absorption of dermal interstitial fluid. The underlying polymer structure exhibits a hydrophilic framework capable of extensive aqueous absorption within three-dimensional networks. Drug incorporation into hydrogel microneedles follows two distinct strategies: The first approach involves drug deposition at the microneedle base, wherein subsequent skin penetration induces hydrogel expansion through interstitial fluid absorption, establishing controlled-release channels between the capillary network and drug reservoir. The release kinetics remain governed by the hydrogel’s cross-linking density, which functions as a molecular diffusion regulator upon swelling [72]. These designs feature integrated drug reservoirs, enabling enhanced payload capacity, though extended application periods that facilitate a larger dose administration. Unlike conventional dissolving microneedles, these arrays maintain removal capability without residual dermal polymer deposition [68]. The second approach involves direct drug–polymer integration throughout the microneedle matrix and substrate, enabling drug release through physiological fluid-mediated swelling mechanisms. These swelling microneedles demonstrate exceptional morphological versatility, facilitate sterilization protocols, and ensure complete dermal removal. The applications of swelling microneedles span interstitial fluid extraction [104], small molecule delivery [105], antibody administration, oncological interventions [106], and wound management [107].

The fundamental composition of swelling microneedles contains cross-linked hydrogel networks, prominently featuring gelatin methacryloyl and methacrylated hyaluronic acid [108]. Upon skin insertion, these needles demonstrate rapid volumetric expansion through fluid uptake, facilitating drug release while maintaining structural integrity for subsequent removal. The intrinsic capability of these systems to extract and retain interstitial fluid (ISF) positions, and swelling microneedles as promising diagnostic tools, leveraging leverages ISF’s rich biomarker content [109]. The swelling properties remain precisely tunable through fabrication-controlled cross-linking density. Comparative analyses reveal the limited exploration of swelling microneedles in vaccination applications relative to alternative designs. Notable research by Courtenay et al. introduced innovative swelling microneedles incorporating Gantrez S-97 and poly(ethylene glycol), combined with ovalbumin-containing lyophilized reservoirs. Immunological studies demonstrated comparable IgG responses between swelling and dissolving microneedles, though dissolving needles exhibited enhanced anti-ovalbumin specific IgG titers, attributed to simultaneous polymeric adjuvant and ovalbumin release [110]. Current vaccination applications utilizing swelling microneedles warrant extensive investigation.

### 4.6. Porous Microneedles

**Figure 5:**
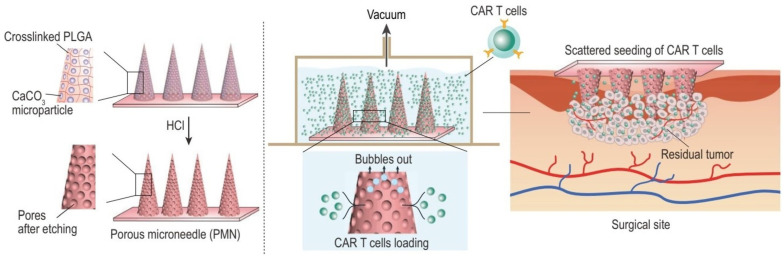
Representation of CAR T cell-incorporated porous microneedles. Image reprinted with permission from [116], 2022, *National Science Review*.

Recent advances in transdermal drug delivery systems have highlighted the exceptional potential of porous microneedles, whose integrated micro/nanoscale channel networks facilitate enhanced therapeutic payload administration (Figure 5). The fabrication of these sophisticated devices utilizes an array of biocompatible substrates, including metallic compounds, ceramic materials, and polymeric matrices [70]. Metallic substrates, particularly titanium and stainless steel alloys, have emerged as predominant materials in porous microneedle development. The characteristic porosity is achieved through the thermal sintering of metallic particulates, yielding interconnected void networks [111]. Analogous fabrication methodologies extend to bio-ceramic substrates, notably alumina, where sintering protocols and anodization techniques generate desired porous structures [112]. Despite superior mechanical robustness and precise porosity control exhibited by metallic and bio-ceramic materials, polymers have garnered substantial interest owing to streamlined manufacturing protocols that circumvent complicated micromachining requirements. Polymeric materials include poly glycidyl methacrylate, poly lactic-co-glycolic acid, polydimethylsiloxane, and cellulose acetate matrices [113,114,115]. The inherent porous structure confers substantial drug-loading capacity for pharmaceutical and vaccine administration. These arrays function as an independent drug delivery system, wherein therapeutic agents, in either liquid or solid states, occupy the porous network before skin permeation post-insertion.

Recent investigations demonstrate remarkable vaccine delivery efficacy through porous microneedles. Notably, researchers have developed cellulose acetate-coated porous arrays utilizing a nonsolvent-induced phase separation methodology, achieving optimal pore dimensions and density. These needles exhibited exceptional vaccine delivery performance and elicited robust immunological responses in preclinical studies [115,117]. Nevertheless, widespread industrial adoption and commercialization face persistent challenges, primarily stemming from increased material costs and complex, temporally intensive multi-step manufacturing protocols [70].

### 4.7. Advantages of Microneedle-Based Vaccination

Transdermal microneedles demonstrate substantial superiority over conventional needle-and-syringe vaccination methodologies. These innovative devices maintain minimal microbial burden while facilitating convenient, atraumatic administration through reduced neural stimulation and diminished nociception. The administration protocol eliminates hemorrhage and pathogenic introduction while offering a simple deployment suitable for both healthcare professionals and self-administration, thereby expanding vaccination accessibility in resource-limited regions [118]. This enhanced user experience promotes vaccination compliance, particularly crucial for multi-dose immunization regimens [119,120].

The design of microneedles minimizes occupational safety risks, including needlestick accidents and cross-contamination events, while streamlining disposal procedures. The operational advantages, coupled with potential dose optimization, superior immunogenic responses, and enhanced vaccination coverage, contribute to substantial cost reduction in immunization programs [49]. Furthermore, microneedles demonstrate broad compatibility across vaccine categories, including live, inactivated, and subunit formulations [121,122], generating comparable or superior immunogenic responses relative to traditional vaccination methods [123,124].

Microneedle technology significantly enhances vaccine penetration, enabling precise administration into the vascularized dermis and epidermis, thereby activating antigen-presenting cells and inducing robust antigen-specific antibody production [56]. Clinical investigations demonstrate that intradermal vaccine delivery via microneedles generates immune responses equivalent to conventional subcutaneous or intramuscular administration routes [125]. From a pharmaceutical stability perspective, microneedle-mediated vaccine delivery presents distinctive advantages. The solid-state formulation of microneedle vaccines exhibits enhanced thermal stability compared to liquid preparations. The combination of the dry vaccine state and stabilizing excipients significantly improves thermostability [125]. These characteristics eliminate reconstitution requirements and minimize cold-chain dependencies [126], establishing microneedles as an advanced alternative to traditional injection methods [127].

The application methodology varies based on array configuration, employing either direct pressure or specialized applicators designed to overcome cutaneous viscoelastic and mechanical properties. The incorporation of vaccines through coating or direct formulation within microprojections enables enhanced stability profiles. These technological attributes deliver multiple operational advantages, including the elimination of needlestick injuries, needle-associated anxieties, cross-contamination risks, and requirements for the specialized preparation or trained personnel. The cumulative benefits of microneedle technology, including dose optimization, enhanced stability profiles, reduced cold-chain requirements, broad vaccine compatibility, and improved vaccination accessibility, particularly in resource-constrained environments, position these devices as a superior alternative to conventional injection methodologies [83,87,128,129].

### 4.8. Barriers to Microneedle-Based Vaccination

Recent advances have established microneedle vaccination as an innovative therapeutic strategy that addresses numerous limitations associated with conventional vaccine administration routes. This approach demonstrates remarkable advantages, including enhanced thermostability, dose-sparing effects, and improved vaccination accessibility. Nevertheless, substantial obstacles persist regarding clinical translation and scale manufacturing processes.

The crucial concern centers on vaccine safety protocols. The manufacturing and storage of both whole-antigen and subunit microneedle vaccines necessitate stringent quality control measures. The sterilization process presents a particularly complex challenge. General sterilization methodologies, encompassing dry heat, autoclave processes, and gamma irradiation, typically compromise vaccine integrity and biological activity [130]. While phthalene-based treatments and electron beam irradiation show promise for microneedle sterilization, these approaches have not achieved complete sterility [131]. The development of suitable sterilization protocols remains a critical prerequisite for commercial scale-up and regulatory approval.

The inherent immunological characteristics of skin tissue raise additional safety considerations. The remarkable immunocompetence of skin tissue, while advantageous for vaccine delivery, presents challenges regarding local reactions. Clinical observations demonstrate transient erythematous responses following microneedle insertion [132]. These dermatological complications warrant a rigorous safety assessment prior to clinical investigations.

Vaccine stability presents additional complexities. Although dissolving microneedle fabrication typically employs moderate temperature conditions (25–40 °C), these parameters restrict applications for certain vaccine formulations. Notably, recombinant protein antigens exhibit marked instability during extended exposure to ambient conditions [133]. Moreover, specific manufacturing processes, particularly photolithography and wire-drawing techniques, necessitate elevated temperatures approaching 100 °C, potentially compromising vaccine integrity [134]. Thermolabile antigens demonstrate heightened sensitivity to these processing conditions.

A limited drug-loading capacity constitutes a significant technological barrier. The majority of microneedle systems demonstrate restricted loading capabilities, with hollow microneedles providing continuous or demand-based administration as notable exceptions [135]. The mechanical compression of dermal tissue frequently occludes hollow microneedle lumens post-insertion. Mechanical compression-induced occlusion can occur through various mechanisms, including tissue coring during insertion and subsequent compressed tissue plugging of the microneedle lumen. Despite enhanced dermal penetration, successful vaccination heavily depends on passive diffusion kinetics. Achievement of immunological threshold doses through passive mechanisms often results in variable antibody responses [136]. This fundamental limitation represents a persistent challenge in microneedle vaccine development.

Material efficiency during manufacturing warrants consideration. Significant vaccine losses occur during microneedle fabrication processes, particularly in coated and dissolving microneedles requiring precise molding and desiccation parameters [137]. Optimization strategies, including extended delivery durations and minimized solvent usage, may mitigate product losses during administration [138]. While microneedle vaccination demonstrates considerable potential as an emerging immunization strategy, multiple technological challenges must be addressed prior to clinical implementation and commercial manufacturing. These challenges include safety considerations, sterilization protocols, stability requirements, payload limitations, and manufacturing efficiency.

## 5. Microneedle Applications for Different Types of Vaccines

The commencement of microneedle vaccination emerged through the pioneering investigations of Mikszta and colleagues [58], who demonstrated the feasibility of transdermal vaccine administration. Subsequently, diverse vaccine types have been adapted for microneedle-mediated delivery, each exhibiting distinctive characteristics and therapeutic advantages. Live-attenuated formulations include attenuated viral or bacterial strains capable of eliciting robust immunological responses while maintaining an acceptable safety profile. Nevertheless, these formulations present inherent risks of phenotypic reversion and necessitate precise thermal regulation above 8 °C [139]. Microneedle administration has demonstrated remarkable advantages, yielding enhanced immunogenicity compared to conventional subcutaneous administration, coupled with superior thermal stability—notably maintaining 90% immunological potency following 4-month storage at 40 °C [140]. Inactivated vaccine preparations, generated through chemical or physical pathogen inactivation [141], although exhibiting reduced immunogenicity compared to live-attenuated vaccines, offer enhanced safety profiles while maintaining the capacity to generate robust immune responses through prime-boost strategies or adjuvant incorporation. Experimental evidence demonstrates that microneedle-administered inactivated vaccines preserve stability for three weeks at 50 °C, whereas conventional formulations experience 40% antigenic degradation within one week [142]. Pathogen component vaccines comprise multiple subcategories. DNA-based immunizations utilize antigen-encoding plasmid vectors [143], offering thermal stability and scalable manufacturing advantages [144]. RNA vaccines facilitate antigen expression through delivered mRNA sequences [145], incorporating protective lipid nanoparticle technologies [146]. Protein subunit formulations employ purified pathogenic proteins, necessitating adjuvant incorporation and multiple administrations. Virus-like particles (VLPs) represent non-infectious viral structural assemblies that maintain native viral features while lacking genomic material [147].

Global applications of microneedle vaccine delivery have demonstrated efficacy across diverse pathogenic targets. Measles vaccination achieved comparable neutralizing antibody levels to standard protocols [148]. Polio immunization elicited robust antibody responses: Rhesus macaque immunization studies demonstrated comparable neutralizing antibody titers between the microneedle patch and conventional intramuscular administration for inactivated poliovirus vaccine serotypes 1 and 2 [149]. Tuberculosis studies revealed significantly enhanced antibody production compared to conventional delivery systems. Low-dose immunization of the Ag85B DNA vaccine (4.2 μg) yielded comparable responses between the microneedle patch and intramuscular administration. In contrast, high-dose delivery (12.6 μg) via microneedles elicited superior humoral immunity compared to IM injection [150]. HIV investigations demonstrated increased bone marrow plasma cell populations and enhanced germinal center responses [151]. Influenza studies exhibited equivalent or superior immunological responses with marked dose-sparing benefits [152]. Research outcomes of microneedle-mediated COVID-19 vaccination remain promising, with selected studies demonstrating 50-fold greater antibody titers compared to conventional administration routes [126].

## 6. Technical Considerations

Recent advances in microneedle-based vaccination have demonstrated remarkable efficacy in pre-clinical investigations, yielding immunological responses that match or surpass traditional parenteral administration routes. Microneedle-based vaccination, while highly promising, does face specific limitations in antigen delivery. Microneedles can effectively deliver small molecules and certain proteins, but larger antigens or complex biological structures may present challenges due to the physical constraints of the microscale needles. This limitation particularly affects the delivery of whole-cell vaccines or large protein complexes. Furthermore, the limited surface area and volume of microneedles can constrain the maximum deliverable dose. While this has been partially addressed through innovative formulation strategies and array designs, certain vaccines requiring larger doses may still necessitate traditional delivery methods.

Human clinical trials remain nascent, with only limited completed studies despite the exponential growth in research activities. The current limited clinical development of microneedle vaccination stems from several key factors. The production of microneedle patches requires sophisticated manufacturing processes and quality control measures to ensure consistent needle geometry, sterility, and antigen stability. Scaling these processes for commercial production presents significant technical and economic challenges. In addition, as a relatively new delivery platform, microneedle-based vaccines require extensive safety and efficacy data to meet regulatory requirements. The pathway to regulatory approval is more complex compared to traditional needle-based formulations, given the novel nature of both the delivery system and associated manufacturing processes. Manufacturing scalability presents a persistent challenge for clinical translation [153]. The World Health Organization mandates that microneedle-delivered vaccines must demonstrate stability profiles meeting or exceeding Vaccine Vial Monitor category type 7 specifications. Substantial technological obstacles persist in the development pipeline, particularly regarding the preservation of liquid vaccine formulation integrity and manufacturing process optimization. The inability to employ conventional sterile filtration methods, coupled with potential antigen degradation during radiation sterilization, necessitates aseptic manufacturing protocols. Furthermore, regulatory frameworks, specifically FDA 21 CFR 3.2, classify microneedle-based vaccines as combination products, mandating comprehensive documentation for both immunogenic and delivery components. Commercial entities have initiated extensive development programs for microneedle-based prophylactic strategies targeting SARS-CoV-2, various infectious diseases, and malignancies, demonstrating the particular value for rapid pandemic response capabilities [154]. While these developments herald promising therapeutic applications, further research endeavors remain essential to overcome existing technological limitations.

### 6.1. Safety

The critical consideration in microneedle vaccine administration remains recipient safety and tolerability. Clinical investigations demonstrate skin barrier restoration within 48 h following the application, with the complete resolution of erythematous symptoms observed over a 7-day period in immunocompetent subjects. Nevertheless, a comprehensive evaluation of skin barrier disruption and associated infection susceptibility warrants further investigation, particularly among geriatric and immunodeficient populations [87,155]. Although the device’s structural integrity and potential tissue trauma require a thorough assessment, current methodological approaches for quantifying penetration dynamics and tissue disruption remain suboptimal [11]. Material selection substantially influences safety parameters. Silicon-based and vitreous silica microneedles elicit concerns regarding mechanical failure and skin irritation, whereas polymeric alternatives offer enhanced safety profiles through biodegradable and biocompatible formulations [156]. Despite predominantly mild adverse events associated with microneedle-mediated vaccination, intradermal delivery demonstrates an increased frequency of localized reactions, including erythema and induration [65]. Regulatory guidance from the US FDA mandates an early-stage consideration of microneedle sterilization protocols, with sterilization requirements determined by multiple parameters, including target tissue characteristics, penetration specifications, application duration, and recipient demographics [157]. Vaccines incompatible with conventional sterilization methodologies necessitate aseptic manufacturing processes, substantially increasing production expenditure [41]. Pandemic scenarios may warrant supervised self-administration protocols, contingent upon maintaining proper application techniques. Despite promising potential for enhancing global vaccine accessibility, microneedle technology necessitates extensive preclinical and clinical validation prior to widespread deployment [41].

### 6.2. Efficacy

Nanocarrier-mediated vaccine delivery through microneedles represents a pioneering approach in immunization technology. Specifically, cationic nanoparticulate systems demonstrate remarkable capabilities in enhancing vaccine stability and facilitating cytoplasmic translocation, particularly in transcutaneous administration [158]. The implementation of these vaccine-encapsulated cationic nanocarriers in conjunction with microneedles elicits superior immunological outcomes compared to conventional administration routes. While transcutaneous and intramuscular delivery exhibited equivalent systemic immunity for cationic nanoparticle-DNA complexes, microneedle-mediated transcutaneous administration uniquely induced robust mucosal immunity and achieved optimized Th1/Th2 balance [159]. Nevertheless, comprehensive clinical validation remains essential to establish the safety profile and therapeutic efficacy of these integrated delivery systems [95]. The incorporation of immunopotentiators serves as a crucial strategy for enhancing vaccine-induced immunity. Recent adjuvant development involves diverse molecular and genetic modulators, including polyinosinic: polycytidylic acid, cytosine-phosphorothioate-guanine oligodeoxynucleotides, and cytokine-encoding plasmids. Classical adjuvant formulations, notably aluminum-based compounds, and MF59, demonstrate a substantial capacity to amplify DNA vaccine-mediated antibody responses [160]. The selection of appropriate adjuvants for microneedle array patches demands thorough consideration, as conventional aluminum-based and oil-in-water emulsion adjuvants present compatibility challenges in microneedle coating processes [41].

Complementing chemical adjuvantation, physical enhancement strategies demonstrate remarkable potential. Research reveals that microneedle-induced localized cellular necrosis within the skin microenvironment functions as an endogenous immunostimulant [161]. Nonetheless, substantial research efforts remain necessary to optimize and standardize microneedles for universal immunization applications. The absence of harmonized protocols, standardized animal models, and unified efficacy parameters poses significant challenges in preclinical evaluation [87]. Moreover, comprehensive investigation continues to elucidate microneedle-mediated immunological mechanisms and subsequent skin immune responses [41].

The robustness of immune responses represents a critical determinant in evaluating microneedle-based vaccination efficacy. Experimental evidence validates the immunological competence of microneedle-mediated vaccine delivery, with numerous designs exhibiting remarkable dose-sparing capabilities [95]. These systems achieve comparable or enhanced immunogenicity relative to traditional parenteral administration, wherein specific microneedle configurations generate sufficient immune responses utilizing substantially reduced antigen doses [162]. This dose-optimization phenomenon yields significant advantages, including the conservation of immunogenic material and potential single-dose vaccination protocols [163].

### 6.3. Acceptability

The widespread adoption of microneedle-based immunization involves multiple determinants: personal predispositions, healthcare provider endorsements, familial influences, perceived operational simplicity, therapeutic efficacy, physiological tolerability, and administration verification protocols. Regulatory authorization emerges as an essential factor governing public acceptance [38]. Recent investigations reveal a significant patient preference for microneedle-based vaccination methods compared to conventional hypodermic delivery systems. The prospect of self-administration demonstrates particular promise, exhibiting enhanced vaccination compliance rates while potentially yielding significant economic benefits through reduced healthcare personnel dependencies [164].

Nevertheless, several impediments to widespread implementation persist, encompassing dermatological complications such as erythema and pruritus [65], limited public familiarity, uncertainties regarding practical applications in pediatric populations, potential immunological adverse reactions, and challenges in confirming successful vaccine delivery. Although microneedle-mediated vaccination demonstrates favorable safety profiles with manageable local responses and can generate equivalent or superior immunological responses compared to traditional parenteral administration, broad clinical implementation remains constrained predominantly by suboptimal immunogenicity [165]. These limitations stem from fundamental challenges in vaccine payload capacity, molecular stability, and transdermal delivery efficiency [165,166,167]. Resolution of these technical barriers through comprehensive preclinical investigations remains imperative prior to advancing clinical evaluation protocols.

### 6.4. Cost-Effectiveness

The implementation of conventional hypodermic immunization protocols necessitates substantial financial investment involving sterile manufacturing environments, sterilization procedures, lyophilization processes, complex logistic networks, temperature-controlled distribution chains, injection apparatus, sterile water supplies, and specialized healthcare personnel [168]. In contrast, the administration of vaccines via microneedles represents an economically advantageous alternative, circumventing the requirements for cleanroom manufacturing facilities, cold-chain maintenance, and medical professionals. Microneedle technology offers significant cost advantages across the entire vaccination value chain. These benefits include the elimination of cold-chain requirements, reducing storage and transportation costs, minimized medical waste disposal expenses, reduced healthcare personnel training and deployment costs due to self-administration capability, a lower risk of needle-stick injuries, and the potential for mass production scale-up, which could reduce manufacturing costs to competitive levels. Furthermore, ongoing technological advancements and economies of scale in microneedle manufacturing are expected to progressively decrease production costs, enhancing overall cost-effectiveness. Although economic viability correlates with immunization acceptance, vaccine potency, and operational expenditures, the predominant costs associated with conventional vaccination stem from administration procedures rather than manufacturing processes [169]. Microneedle-based delivery systems enhance vaccination compliance and population coverage while reducing the incidence of transmissible diseases, consequently diminishing hospitalization frequencies and mortality rates [170,171]. Economic analyses demonstrate remarkable cost reductions through microneedle vaccination strategies, with projected savings approximating 40% per vaccination event compared to conventional methodologies [168]. These innovations present opportunities for substantial financial benefits, potentially generating savings of USD 950 million for third-party payers and USD 2.6 billion for society during influenza seasons [172].

### 6.5. Applicators and Wear Times

Transdermal drug delivery encounters substantial physiological barriers at the stratum corneum, impeding the efficient penetration of pharmaceutical agents and immunological preparations into the epidermis and superior dermis, where immune-competent cells predominantly reside [54]. The implementation of microneedle-based immunization necessitates precise skin penetration, demanding application devices to ensure reproducible delivery outcomes. Essential engineering parameters for applicator optimization include uniform force distribution across diverse dermatological conditions, precise penetration depth control, insertion velocity, requisite mechanical durability, operational simplicity, and sustained structural integrity during storage conditions [173]. Skin’s viscoelastic properties necessitate the consideration of microneedle insertion velocity. Higher insertion speeds can reduce the deformation of viscoelastic tissue, facilitating more consistent penetration depths. Conversely, slower insertion velocities may result in increased tissue deformation and variable penetration profiles due to the skin’s time-dependent mechanical response. Administration devices are available in both manual and automated configurations, accommodating single-patient or multiple-recipient applications.

The duration requirements for microneedle administration exhibit considerable variation based on structural designs: coated and hollow microneedles demand short application periods ranging from instantaneous to 120 s, whereas dissolving and swelling microneedles necessitate extended contact durations spanning 20 min to 24 h for complete drug release. Multiple pharmaceutical firms have engineered proprietary application systems specific to their microneedle products [41]. Prior to commercial deployment, microneedle technologies must address multiple critical parameters, such as comprehensive safety assessments, thermal stability profiles, reproducible administration protocols, disposal methodologies, economic feasibility, and regulatory compliance. Manufacturing scalability while maintaining sterile conditions represents the critical challenge. Various technological challenges emerge for specific microneedle types: dissolving microneedles face challenges in a sterile matrix formation and solidification processes, while coated needles encounter difficulties in achieving efficient vaccine deposition on microstructures. Engineering innovations must establish cost-competitive manufacturing processes relative to conventional hypodermic delivery systems, while regulatory frameworks require precise definitions for individual microneedle systems [41].

### 6.6. Microneedle Dimensions

The dimensional parameters of microneedles play a crucial role in determining skin penetration efficiency and drug delivery outcomes. Geometric configurations (including cylindrical, conical, volcano-shaped, and tapered geometries) and tip dimensions significantly influence penetration mechanics, whereby metallic and silicon-based constructs demonstrate superior insertion characteristics compared to polymeric alternatives [54]. In vaccination applications, microneedles exhibiting tip diameters below 15 µm facilitate optimal access to the Langerhans cell-enriched subdermal region [174]. The longitudinal dimension represents a critical design parameter—microneedles extending beyond 4 mm risk mechanical deformation and potentially interface with pain-sensitive, poorly vascularized adipose regions, presenting substantial limitations for therapeutics requiring systemic distribution [174]. Alternatively, microneedles of insufficient length penetrate solely into the epidermal layer, thereby constraining drug diffusion and subsequent bioavailability. The vertical dimension of microneedles fundamentally determines therapeutic payload capacity through modifications in surface area, tip dimensions, and the internal channel diameter [175]. Although immunization protocols generally demand reduced dosages compared to parenteral administration, primarily attributed to microneedles’ dose-reduction capabilities via Langerhans cell activities [126], a standardized microneedle structure remains elusive due to the heterogeneity in therapeutic agents, pathological conditions, and skin variability among individuals. Such dimensional diversity requirements pose notable challenges for commercial scale-up and clinical implementation [11].

### 6.7. Microneedle Manufacturing

Several companies have been developing microneedle-based vaccination products (Table 1). Manufacturing optimization and cost-effectiveness remain crucial challenges in microneedle array production. Although substantial initial capital investment in equipment and fabrication infrastructure is necessary, the projected operational expenses demonstrate favorable economics compared to conventional injection systems [176]. Despite the apparent cost advantages of polymer-based molding techniques, the scalability of processing systems encounters significant impediments, predominantly due to master template dependencies and complex multi-phase filling protocols.

The sterilization technique presents a significant technical barrier in microneedle device development, particularly for vaccine-incorporated arrays, where conventional filtration methodologies are inadequate. This limitation necessitates the implementation of sophisticated aseptic manufacturing environments to preserve vaccine sterility and immunogenic properties [19]. Thermal management during fabrication emerges as a critical parameter, as the drying process generates heat that potentially compromises thermolabile antigens. Environmental factors, including temperature fluctuations, freeze–thaw cycles, pH variations, and photochemical exposure, significantly influence vaccine stability [139], with particular implications for mRNA-based and viral vector vaccines. Storage specifications, packaging requirements, and quality assurance protocols introduce additional complexities. The incorporation of moisture-barrier packaging materials becomes essential, albeit with associated cost implications [16]. Manufacturing companies must establish comprehensive pharmaceutical quality systems aligned with Good Manufacturing Practice standards and Quality Risk Management protocols.

### 6.8. Regulation

The US FDA classifies microneedle arrays as drug-device combination products under regulatory statute 21 CFR 3.2e, necessitating comprehensive oversight [177]. The regulatory framework mandates a thorough evaluation of safety and efficacy parameters for both discrete components and the integrated therapeutic system, in accordance with 21 CFR Part 4 Subpart A: Section 4.4 (b). The regulatory landscape presents unique challenges, as microneedle-based vaccine systems occupy a dual classification as biological products and mechanical devices, necessitating compliance with regulatory requirements for individual components and the integrated delivery system [19,67]. Critical regulatory considerations cover the establishment of current good manufacturing practice (cGMP) protocols and sterilization methodologies alongside the development of comprehensive guidelines governing patient-administered therapeutic applications [178].

### 6.9. Sustainability

Recent pharmaceutical innovations mandate environmental sustainability; nevertheless, conventional hypodermic delivery systems remain exempt from these requirements owing to critical cross-contamination considerations, necessitating single-administration protocols. The emergence of microneedle technology introduces novel sustainability obligations that must be harmonized with therapeutic efficacy parameters. Silicon-based microneedles demonstrate substantial ecological limitations attributed to stringent material purity specifications and water-intensive fabrication methodologies [179]. In contrast, biodegradable and dissolvable microneedle systems present promising alternatives for achieving zero-waste pharmaceutical administration methods [122].

## 7. Pre-Clinical Studies of Microneedle-Based Vaccination

Microneedle technology demonstrates remarkable versatility across various vaccine types, from conventional protein-based vaccines to advanced mRNA formulations [83,180]. Recent advances in microneedle engineering have yielded significant breakthroughs in vaccine stability and efficacy. Notably, mRNA vaccines incorporated into microneedle patches maintain bioactivity for at least 6 months at room temperature, with single-patch delivery achieving therapeutic doses through efficient loading and dissolution kinetics [83]. This stability enhancement also extends to traditional vaccines, exemplified by a five-in-one microneedle patch incorporating Diphtheria, Tetanus, Pertussis, Hepatitis B, and Haemophilus influenza type b vaccines, which preserved antigenicity for 12 months at 25 °C [180].

The immunological advantages of microneedle-based vaccination stem from strategic targeting of the skin’s immune system. Dissolving microneedle arrays have demonstrated particular promise when combined with advanced delivery systems. The incorporation of poly(D, L-lactic-co-glycolic acid) nanoparticles into dissolving microneedles enabled enhanced immune responses, though optimal formulation remains crucial for maintaining particle integrity and achieving efficient skin penetration [181]. Similarly, egg microneedles loaded with influenza vaccine have shown superior protective efficacy compared to intramuscular administration, even at reduced doses [182].

The application of microneedle technology extends beyond traditional vaccinations into cancer immunotherapy. Novel approaches include ice microneedle arrays for delivering living tumor cell vaccines with sustained granulocyte-macrophage colony-stimulating factor secretion [183] and photothermal ultra-swelling microneedle patches achieving remarkable swelling ratios exceeding 2000% for efficient tumor antigen delivery [184]. These systems provided enhanced dendritic cell recruitment and CD8^+^ T cell infiltration in tumors compared to subcutaneous administration [183]. Furthermore, microneedles combined with Peptide-coated Conditionally Replicating Adenoviruses (PeptiCRAds) have achieved complete tumor rejection in pre-vaccinated subjects through enhanced cDC1 population stimulation in lymph nodes [185].

Formulation optimization remains central to microneedle vaccine development. The integration of liposomes into dissolving microneedle patches has shown particular promise for Hepatitis B vaccination, generating significantly higher anti-HBsAg IgG antibody levels compared to conventional subcutaneous injections [186]. These advances in formulation technology, combined with innovative delivery mechanisms, demonstrate the potential of microneedles to improve vaccine administration across a broad spectrum of applications.

## 8. Clinical Studies of Microneedle-Based Vaccination

Extensive clinical investigations have demonstrated the translational potential of microneedles for transcutaneous vaccine administration. The progression of microneedle-facilitated immunization research has witnessed substantial advancement from preclinical studies to human clinical trials. A comprehensive analysis revealed that, between 2012 and 2022, twenty-six clinical studies were initiated globally, with eighteen reaching successful completion [95]. To establish the current landscape of microneedle-enabled vaccination strategies, systematic searches were conducted on the International Clinical Trials Registry Platform Search Portal, utilizing the search terms “microneedle” and “vaccine”, with time constraints focusing on investigations within the preceding decade (Table 2). The primary endpoints of these clinical investigations included safety profiles, immunological responses, and patient acceptability metrics. A notable evolution in microneedle design has emerged, transitioning from predominantly hollow structures toward diverse structural configurations.

## 9. Combination with Other Technologies

The implementation of transdermal immunotherapeutic approaches encounters significant impediments attributed to the inherent barrier functions of skin tissue. Technological innovations synergistically integrated with microneedles have emerged to enhance dermal penetration through strategic circumvention of the stratum corneum [70]. These advanced methodologies are photodynamic therapy (PDT), low-frequency sonophoresis (LFS), iontophoretic delivery, and electroporative techniques. The mechanism of PDT [187] leverages photosensitizing compounds activated by wavelength-specific illumination to generate cytotoxic reactive oxygen species, facilitating selective cellular ablation. The integration of PDT with microneedle-based immunotherapy demonstrates remarkable potential for localized oncological interventions [70]. LFS technology employs ultrasonic waves in the low-frequency spectrum (20–100 kHz) to transiently disrupt stratum corneum integrity through cavitation-induced microjet formation [188]. The concurrent application of microneedles and LFS yields optimized cutaneous permeability while minimizing collateral tissue damage [189]. Iontophoretic administration employs controlled direct or alternating current (0.5 mA/cm^2^) to facilitate molecular transport via electrorepulsive forces [190]. The incorporation of microneedles enhances macromolecular protein delivery and enables precise dosing control through current modulation [70]. Electroporative delivery employs the application of high-voltage electrical impulses (>50 V, 1–100 ms) to generate transient aqueous channels within the stratum corneum [191]. The synergistic combination with microneedles achieves the simultaneous penetration of cutaneous and cellular barriers while reducing tissue damage and neural stimulation [192]. This integrated approach demonstrates particular efficacy for the delivery of high-molecular-weight therapeutic agents, including insulin and nucleic acids. These diverse technological platforms, when strategically combined with microneedle systems, exhibit substantial promise for advancing transdermal drug delivery, potentially offering complementary advantages. Future investigations should prioritize the development of cost-effective integration strategies to optimize delivery efficiency.

## 10. Conclusions

The advent of microneedle technology in the late 1990s has revolutionized transdermal drug delivery systems. These innovative systems leverage biodegradable polymeric materials with customizable physicochemical properties, facilitating controlled immunogen release kinetics for enhanced vaccination strategies. The remarkable efficacy of microneedle-based delivery stems from the precise targeting of cutaneous immune components, specifically Langerhans cells and dermal dendritic cells, which orchestrate robust immunological responses against viral and bacterial pathogens. Microneedles demonstrate substantial superiority over conventional hypodermic administration routes. These advantages include reduced nociception, enhanced safety profiles, superior cost-effectiveness through dose-sparing effects, and enhanced stability with diminished cold-chain requirements. The self-administration of microneedles, requiring minimal healthcare expertise, renders this technology particularly advantageous for mass immunization campaigns during pandemic scenarios. Moreover, sustained-release polymer-based microneedle systems potentially eliminate the necessity for multiple immunization doses. Although microneedle technology holds transformative potential for global vaccination initiatives, particularly in resource-limited settings, several technical issues warrant consideration. Critical challenges include scale-up manufacturing capabilities, economic feasibility assessments, aseptic processing protocols, and quality control standardization. The successful commercialization of affordable microneedle-based vaccines necessitates comprehensive development strategies, including formulation optimization, validation protocols, and regulatory compliance. Notwithstanding these challenges, microneedle vaccination technology presents unprecedented opportunities to revolutionize global immunization practices and enhance pandemic preparedness.

## 11. Future Perspectivesof Microneedle-Based Vaccination

Immunization protocols remain the essential strategy for mitigating epidemic outbreaks that pose substantial risks to public health. Although nucleic acid-based vaccines have garnered significant attention, particularly exemplified by COVID-19 prophylactics, due to their economic feasibility and precise targeting capabilities, the quest for an optimal vaccination methodology persists [95]. Microneedles demonstrate particular relevance in addressing global health disparities. Microneedle systems offer substantial advantages for geographically isolated communities, geriatric populations, mobility-impaired individuals, needle-phobic pediatric patients, and regions lacking temperature-controlled supply chains. Nevertheless, several obstacles require attention, including manufacturing scalability challenges, physiological considerations regarding dermal variability, and regulatory compliance requirements. Recent technological advances include additive manufacturing methodologies, integrated diagnostic capabilities, and enhanced sterilization protocols [67]. Recent developments in 3D printing technologies, particularly two-photon polymerization and microstereo-lithography, present noticeable opportunities for microneedle development. Moreover, emerging high-throughput 3D printing shows promise for industrial-scale production, potentially improving microneedle manufacturing economics. The convergence of 3D printing with smart materials could enable next-generation responsive microneedles with programmable dissolution kinetics and enhanced vaccine stability. Recent breakthroughs in manufacturing technologies and materials science have significantly improved production scalability and cost-effectiveness. Growing investment in microneedle research and development by major pharmaceutical companies and increased regulatory agency engagement suggest an approaching inflection point for commercial viability. Moreover, the COVID-19 pandemic has heightened interest in alternative vaccination methods, potentially accelerating the regulatory pathway for microneedle-based delivery systems. Through sustained research investment and technological refinement, market introduction of the microneedle-based vaccine formulation appears feasible within a five-year horizon [41].

## Figures and Tables

**Table 1:** Companies developing microneedle-based vaccination products [19].

Company	Type of Microneedle	Vaccine
3 M (Kindeva)	Coated microneedle	Influenza
	Hollow microneedle	Cancer vaccines
JUVIC	Dissolving microneedle	Scrub typhus
Micron Biomedical	Dissolving microneedle	Inactivated poliovirus vaccine and inactivated rotavirus vaccine Measles
Vaxxas (Nanopatch^TM^)	Coated microneedle array patch	Influenza, COVID-19
Quadmedicine	Dissolving microneedles Coated microneedles	Influenza, Hepatitis B, canine influenza
Vaxess	Dissolving microneedles	Influenza, COVID-19, skin cancer
Raphas	Dissolving microneedles	HPV, polio, Tdap, HBV, IPV, and Hepatitis B
BD Technologies (BS Soluvia)	Stainless steel microneedle	Influenza
Flugen	Metal microneedle	Influenza
Debiotech	Hollow microneedle	COVID-19
Verndari (Vaxipatch)	Stainless steel microneedle	Influenza, COVID-19
Nanopass (MicroJet^TM^)	Silicon microneedle	Influenza, polio, Varicella Zoster, cancers, Hepatitis B, COVID-19
BioSerenTach Inc.	Dissolving microneedle	Vaccine
Sorrento therapeutics (Sofusa^®^)	Nanotopographical imprinted microneedle (Coated microneedles)	Immuno-oncology

**Table 2:** List of completed clinical trials for microneedle-based transdermal vaccination.

NCT Number	Phase	Study Title	Interventions	Microneedle System	Conditions	Sponsor/Collaborators
NCT02995057	NA	Safety Demonstration of Microneedle Insertion	Gold- or silver-coated, or uncoated nickel microneedles	Metal microneedles	Allergic Reaction to Nickel	University of British Columbia
NCT06125717	1	Phase 1 Evaluation of H1 Influenza Vaccine Delivered by MIMIX MAP	Biological: H1 influenza antigen	A MIMIX Microneedle Array Patch (MAP) System	Influenza	Vaxess Technologies
NCT01813604	3	Immunogenicity of Inactivated and Live Polio Vaccines	Group A: Trivalent Oral Polio Vaccine Group B: Bivalent Oral Polio Vaccine Group C: Inactivated Polio Vaccine Group D: fractional IPV (f-IPV) Arm E: f-IPV and bOPV	MicroJet 600 microneedle	Poliomyelitis	Centers for Disease Control and Prevention
NCT03607903	1, 2	Adalimumab Microneedles in Healthy Volunteers	Adalimumab ID Adalimumab SC Saline ID Saline SC	MicroJet 600 microneedle	Pain Injection Site	Centre for Human Drug Research, Netherlands
NCT00703651	2	Study of Inactivated, Split-Virion Influenza Vaccine Administered by Intradermal Route Versus Vaxigrip^®^ in Adults	Biological: Inactivated, split-virion influenza vaccine Trivalent Influenza vaccine, (Inactivated, split-virion vaccine)	Microprojection system	Influenza Orthomyxoviridae Infection Myxovirus Infection	Sanofi Pasteur, a Sanofi Company
NCT04064554	NA	Clinical Study to Evaluate Safety and Immunogenicity of Bacillus Calmette-Guerin (BCG) Delivery Via Novel Micronjet600 Device Compared to Those Via Conventional Needle	Device: Bacillus Calmette–Guerin vaccination with MicronJet600 Device: BCG vaccination with conventional needle	MicronJet 600^®^	Tuberculosis BCG Vaccination	Yonsei University
NCT01049490	NA	Dose Sparing Intradermal S-OIV H1N1 Influenza Vaccination Device	Biological: S-OIV H1N1 vaccine	Micron Jet 600^®^	Influenza Infection	The University of Hong Kong Hospital Authority, Hong Kong
NCT01304563	NA	2010/2011 Trivalent Influenza Vaccination	Biological: TIV 2010/2011 influenza vaccine Biological: INT	Micron Jet	Influenza	The University of Hong Kong Hospital Authority, Hong Kong
NCT01686503	2	Intradermal Versus Intramuscular Polio Vaccine Booster in HIV-Infected Subjects	Drug: IPOL (Sanofi Pasteur) inactivated polio vaccine booster dose	NanoPass MicronJet 600^®^ microneedles device	Polio Immunity	Eastern Virginia Medical School NanoPass Technologies Ltd.
NCT04394689	1 and 2	Measles and Rubella Vaccine Microneedle Patch Phase 1–2 Age De-escalation Trial	Biological: Measles Rubella vaccine (MRV-SC) Biological: MRV-MNP	Dissolving microneedle patch	Measles Rubella Vaccination Healthy	Micron Biomedical, Inc. Medical Research Council Centers for Disease Control and Prevention
NCT02438423	1	Inactivated Influenza Vaccine Delivered by Microneedle Patch or by Hypodermic Needle	Biological: Inactivated influenza vaccine	Dissolvable microneedle patch	Influenza	Mark Prausnitz Emory University Georgia Institute of Technology
NCT02621112	2 and 3	HBV Vaccine in Renal Failure Patients	Biological: Intradermal HBVv with imiquimod Biological: Intradermal HBVv with aqueous cream Biological: Intramuscular HBVv with aqueous cream	Microneedles	Renal Failure	The University of Hong Kong
NCT02329457	2 and 3	VZV Vaccine for Hematopoietic Stem Cell Transplantation	Biological: Zostavax	Microneedles	Varicella Zoster Infection	The University of Hong Kong
NCT01707602	1 and 2	Routes of Immunization and Flu Immune Responses	Biological: INTANZA^®^ 15 Biological: Vaxigrip^®^ Biological: INTANZ^®^ 15 T	Microneedles	Influenza	Assistance Publique–Hôpitaux de Paris Institut National de la Santé Et de la Recherche Médicale, France
NCT03207763	NA	Microneedle Patch Study in Healthy Infants/Young Children	Device: Microneedle Formulation 1 Device: Microneedle Formulation 2	Microneedles	Vaccination Skin Absorption	Emory University Micron Biomedical, Inc.
NCT05315362	2	Establishing Immunogenicity and Safety of Needle-free Intradermal Delivery of mRNA COVID-19 Vaccine	Device: Solid microneedle skin patch	Solid microneedle patch	Vaccination; Infection COVID-19	Leiden University Medical Center
NCT00558649	NA	A Pilot Study to Evaluate the Safety and Immunogenicity of Low-Dose Flu Vaccines	Biological: Flu Vaccine (FLUARIX^®^)	Microneedle Injectors	Influenza, Human	NanoPass Technologies Ltd.
NCT01767324	NA	Site Selection for Intracutaneous Saline Delivery	Device: Injection to deltoid Device: Injection to the forearm Device: Injection to thigh	FLUGEN 101.2 microneedle-based device	Intracutaneous Drug Delivery	FluGen Inc. Accelovance
NCT01039623	NA	Assessment of Safety and Immunogenicity of Intradermal Unadjuvanted Portion of Pandemrix^®^ Via a Microneedles Device With Intramuscular Adjuvanted Pandemrix^®^ as Reference	Biological: Pandemrix^®^ (H1N1 pandemic influenza)	Microneedles	Healthy	Hadassah Medical Organization

## Data Availability

No new data were created or analyzed in this study. Data sharing is not applicable to this article.
